# Comparison of Abbott ID Now, DiaSorin Simplexa, and CDC FDA Emergency Use Authorization Methods for the Detection of SARS-CoV-2 from Nasopharyngeal and Nasal Swabs from Individuals Diagnosed with COVID-19

**DOI:** 10.1128/JCM.00760-20

**Published:** 2020-07-23

**Authors:** Daniel D. Rhoads, Sree S. Cherian, Katharine Roman, Lisa M. Stempak, Christine L. Schmotzer, Navid Sadri

**Affiliations:** a Department of Pathology, University Hospitals Cleveland Medical Center, Cleveland, Ohio, USA; b Department of Pathology, School of Medicine, Case Western Reserve University, Cleveland, Ohio, USA; Boston Children’s Hospital

**Keywords:** COVID-19, nucleic acid amplification, SARS-CoV-2, coronavirus, emergency use authorization, *in vitro* diagnostic, virology

## LETTER

Dozens of *in vitro* diagnostics (IVDs) have received emergency use authorization (EUA) from the U.S. Food and Drug Administration (FDA) for the detection of severe acute respiratory syndrome coronavirus 2 (SARS-CoV-2), but how well these assays perform using clinical specimens has hardly been studied. This study compared the positive percent agreement (PPA) of ID Now (Abbott, Chicago, IL, USA) and Simplexa (DiaSorin, Saluggia, Italy) using a modified CDC method as the reference standard (1). All three methods are used as part of standard of care testing within our hospital system. Ninety-six remnant clinical specimens from April 2020 that tested positive for SARS-CoV-2 using standard of care testing were selected based on convenience and retested using the three methods. Fourteen negative controls (universal transport medium [UTM]) were included to control for carryover contamination. Specimens included 11 supervised self-collected nasal swabs in 2 ml normal saline and 85 provider-collected nasopharyngeal swabs in 3 ml UTM. The online Medcalc tool (https://www.medcalc.org/calc/diagnostic_test.php) was used to determine the exact Clopper-Pearson 95% confidence intervals (95% CI). It is well documented that IVDs for SARS-CoV-2 can return false-negative results in individuals with coronavirus disease 2019 (COVID-19) (2), and our study did not attempt to determine the clinical sensitivity of these assays.

The instructions for use (IFU) were followed for all methods with the exception that nasal swabs in saline were included, but these specimens are not explicitly described as acceptable in all of the assays’ IFU. Other deviations from the IFU for the CDC method include the following: (i) using 7500 Fast instead of 7500 Fast Dx instrument, (ii) using an alternate RNA extraction method (Maxwell RSC instrument with viral TNA kit [catalog no. AS1330; Promega, Madison, WI, USA]), and (iii) interpreting “inconclusive” (one positive target) results as “detected.”

The results are shown in Table 1. The modified CDC assay detected SARS-CoV-2 in all 96 specimens (range of threshold cycle [*C_T_*] values, 11.1 to 39.6). The ID Now assay detected SARS-CoV-2 in 90 of 96 specimens (PPA, 94% [95% CI, 87 to 98%]). The Simplexa assay detected SARS-CoV-2 in 92 of 96 specimens (PPA, 96% [95% CI, 90 to 99%]) (range of *C_T_* values, 9.3 to 34.5).

**TABLE 1:** Positive percent agreement (PPA) of the Abbott ID Now and DiaSorin Simplexa assays for the detection of SARS-CoV-2 was determined using a modified CDC assay as the reference standard

Assay	No. of specimens		PPA (%) (95% CI)
	SARS-CoV-2 detected	SARS-CoV-2 not detected	
Abbott ID Now	90	6	94 (87−98)
DiaSorin Simplexa	92	4	96 (90−99)
Modified CDC assay	96	0	Not applicable

The modified CDC assay detected both N1 and N2 targets in 94 of 96 specimens. In the other two specimens, the modified CDC assay detected only one of its two targets at ≥39 *C_T_*, and SARS-CoV-2 was not detected using ID Now and Simplexa in these two specimens. In six other specimens, SARS-CoV-2 was not detected by only one of the assays; both targets in the modified CDC assay were detected at ≥32 *C_T_* in these six specimens. Strong correlation of *C_T_* values was observed when plotting the modified CDC assay N1 and N2 targets against the Simplexa S and Orf1ab targets (*R*^2^ = 0.89 to 0.91).

In summary, the 95% CIs for the PPA were overlapping for the ID Now and Simplexa assays when using the modified CDC method as the reference standard. The sample size of this study was not large enough to conclude that one of these assays had clearly superior or inferior performance for the detection of SARS-CoV-2 from upper respiratory specimens in liquid transport medium. In addition to an assay’s limit of detection and sensitivity, considerations of other important variables such as turnaround time, complexity, cost, workflow, specimen type and stability, and availability of supplies, reagents, and equipment may influence selection of a health system’s standard of care IVD that is implemented to detect SARS-CoV-2 RNA.
